# Synergic Therapeutic Potential of PEA-Um Treatment and NAAA Enzyme Silencing In the Management of Neuroinflammation

**DOI:** 10.3390/ijms21207486

**Published:** 2020-10-11

**Authors:** Giovanna Casili, Marika Lanza, Michela Campolo, Rosalba Siracusa, Irene Paterniti, Alessio Ardizzone, Sarah Adriana Scuderi, Salvatore Cuzzocrea, Emanuela Esposito

**Affiliations:** 1 Department of Chemical, Biological, Pharmaceutical and Environmental Sciences, University of Messina, 98165 Messina, Italy; gcasili@unime.it (G.C.); mlanza@unime.it (M.L.); campolom@unime.it (M.C.); rsiracusa@unime.it (R.S.); ipaterniti@unime.it (I.P.); aleardizzone@unime.it (A.A.); sarascud@outlook.it (S.A.S.); eesposito@unime.it (E.E.); 2 Department of Pharmacological and Physiological Science, Saint Louis University, Saint Louis, MO 63103, USA

**Keywords:** glioma, neuroblastoma, microglia, oligodendrocytes, neuroinflammation, palmitoylethanolamide

## Abstract

Inflammation is a key element in the pathobiology of neurodegenerative diseases and sees the involvement of different neuronal and non-neuronal cells as players able to respond to inflammatory signals of immune origin. Palmitoylethanolamide (PEA) is an endogenous potent anti-inflammatory agent, in which activity is regulated by N-acylethanolamine acid amidase (NAAA), that hydrolyzes saturated or monounsaturated fatty acid ethanolamides, such as PEA. In this research, an in vitro study was performed on different neuronal (SH-SY5Y) and non-neuronal cell lines (C6, BV-2, and Mo3.13) subjected to NAAA enzyme silencing and treated with PEA ultra-micronized (PEA-um) (1, 3, and 10 μM) to increase the amount of endogenous PEA available for counteract neuroinflammation provoked by stimulation with lipopolysaccharide (LPS) (1 μg/mL) and interferon gamma (INF-γ )(100 U/mL). Cell viability was performed by MTT (3-(4,5-Dimethylthiazol-2-yl)-2,5-Diphenyltetrazolium Bromide) staining, suggesting a protective effect of PEA-um (3 and 10 μM) on all cell lines studied. Western Blot analysis for inflammatory markers (Inducible nitric oxide synthase (iNOS) and cyclooxygenase 2 (COX-2)) was carried out in control and NAAA-silenced cells, highlighting how the concomitant treatment of the neuronal and non-neuronal cells with PEA-um after NAAA genic downregulation is satisfactory to counteract neuroinflammation. These in vitro findings support the protective role of endogenous PEA availability in the neuronal field, bringing interesting information for a translational point of view.

## 1. Introduction

N-acylethanolamine acid amidase (NAAA) is a specific hydrolases that degrades saturated or monounsaturated fatty acid ethanolamides, such as palmitoylethanolamide (PEA) and oleoylethanolamide (OEA) [1]. However, OEA and, to a lesser extent, anandamide (AEA) are hydrolyzed by NAAA, N-acylethanol-amines (NAEs) catabolic enzymes are currently viewed as potential therapeutic targets in which inhibition may increase tissue levels of PEA [2]. The recent researches in strategies to increase the endogenous levels of N-acylethanol-amines (NAEs) has led to the discovery of new selective molecules able to act by inhibiting NAAA [3]. The regulatory role of these enzymes consists in the modulation of substrates available, such as PEA, produced on demand by specific cells to exert protective actions [4]. Specifically, PEA acts on glia and mast cells and, in this context, it may well be that NAAA is physiologically designed to modulate substrate availability.

The pharmacological blockage of these enzymes can lead to disorders, since the substrates, such as PEA, are produced on demand. Through a pharmacological approach, it has been shown that it is more effective to modulate rather than block these enzymes activity. Ultra-micronization process is able to reduce the large particle PEA to micron-sized crystals to enhance dissolution and reduce variability of absorption [5]. Particularly, the ultra-micronization of PEA produces a crystalline structure with higher energy content and lower particle size, contributing to a better distribution and diffusion than the naïve form [6]. Numerous case reports and open-label studies suggest a potential effect of ultra-micronized PEA (PEA-um) on inflammatory diseases [7,8]. PEA is produced and hydrolyzed by microglia, and it downmodulates mast cell activation; because PEA is produced on demand and exerts pleiotropic effects on non-neuronal cells implicated in neuroinflammation, modulating the specific amidases for NAEs could be a way to preserve PEA role in maintaining cellular homeostasis through its rapid on-demand synthesis and equally rapid degradation [9]. Neuroinflammation is a reply from the central nervous system (CNS) and the peripheral nervous system (PNS) to an unbalanced homeostasis. A fundamental principle of neuroinflammation is the existence of extensive lines of communication between the nervous system and immune system; in particular, immune cell-derived inflammatory molecules are critical for regulation of host responses to inflammation; these mediators can originate from various non-neuronal cells, microglia and mast cells, together with astrocytes and possibly also oligodendrocytes, appear to be important sources in neuropathologies [10,11]. Understanding neuroinflammation also requires an appreciation that non-neuronal cell–cell interactions, between both glia and mast cells and glia themselves, are an integral part of the inflammation process. 

In neuroinflammatory process the important players are represented by lipid mediators [12]. Among the naturally occurring lipid signaling molecules, a prominent role is played by NAEs, particularly by PEA, that possesses a powerful neuroprotective and anti-inflammatory power. In fact, PEA normalizes astrocytic function, rules glutamatergic transmission, and confines neuroinflammation; moreover, PEA represents a novel and effective promising treatment for various neurodegenerative diseases, like Alzheimer’s disease (AD) [13]. In terms of substrates, when assessed in vitro, NAAA has a strong preference for saturated NAEs, with PEA being the preferred substrate, although AEA and OEA are also substrates of the enzyme [14]. PEA-um is a substrate for fatty acid amide hydrolase (FAAH) and NAAA, but the regulation, substrate preference, and localization of the enzymes differ.

The aim of the study was to understand if the NAAA silencing, in combination with exogenous PEA-um treatment, increasing the amount of endogenous PEA available, could reduce the in vitro inflammatory process in both neuronal and non-neuronal cells, bringing interesting information for a translational point of view. Particularly, the fact that NAAA has distinct subcellular localization and access to potentially different substrate pools suggests that the concomitant NAAA silencing and PEA-um treatment could lead to a further increase of endogenous PEA levels and potentially stronger neuroprotective effects.

## 2. Results

### 2.1. In Vitro Effects of PEA-um Treatment on Neuronal and Non-Neuronal Cell Lines Viability and Following Inflammatory Stimulation

**Figure 1:**
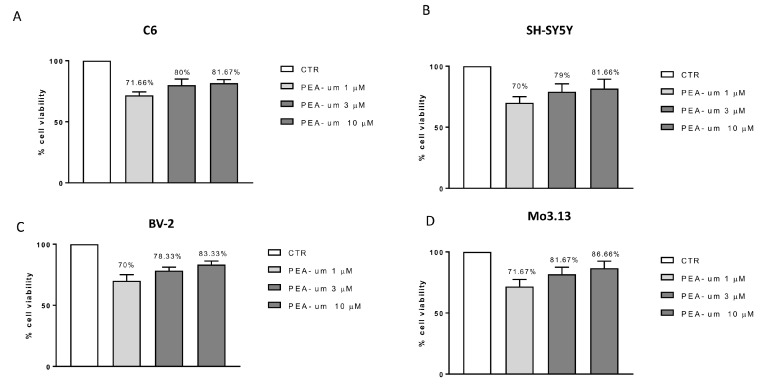
Cell viability evaluation after palmitoylethanolamide (PEA)-um treatment. Cell viability was evaluated using 3-(4,5-dimethylthiazol-2-yl)-2,5-diphenyl tetrazolium bromide (MTT) assay 24 h after treatment with PEA-um (1, 3, and 10 µM) in four cell lines: neuronal SH-SY5Y (**B**), non-neuronal C6 (**A**), BV-2 (**C**), and Mo3.13 (**D**) cells. None of the PEA-um concentrations resulted in a significant cell mortality. Data are representative of at least three independent experiments.

In order to choose the highest PEA-um concentrations with the lowest toxicity, cell viability was performed treating C6, SH-SY5Y, BV-2, and Mo3.13 cells with different concentrations (1, 3 and 10 μM) of PEA-um. In all cell lines tested, the treatment preserved cell viability, resulting in only a 30% vitality reduction, at the lowest concentration (1 µM) (Figure 1A–D). In particular, it was observed that PEA-um treatment, at the concentrations of 3 and 10 µM, behaved in the same way, ensuring a cell viability of about 80% in all cell lines examined (Figure 1A–D).

**Figure 2:**
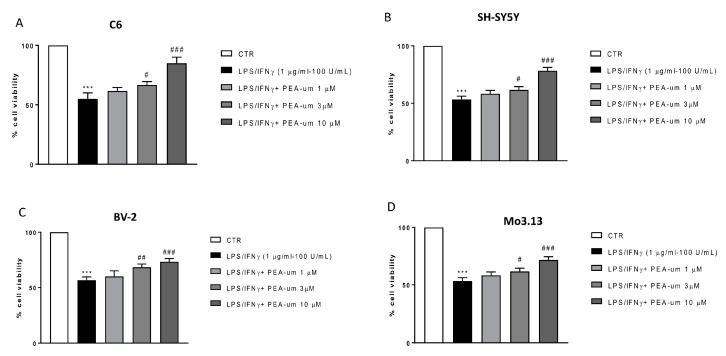
Anti-inflammatory effects of PEA-um in both neuronal and non-neuronal cell lines following LPS/IFN γ-stimulation. Cell vitality was assessed following 24 h treatment with LPS (1 μg/mL) and INF-γ (100 U/mL) and different concentrations (1, 3, and 10 µM) of PEA-um. PEA-um at 3 μM and 10 μM significantly locked damage caused by LPS/INF-γ in all cell lines (**A**–**D**). Particularly, in BV-2 microglial cells, the effects of PEA-um 3 μM were almost comparable to those of treatments at 10 μM (**C**). PEA-um 1 μM is unable to protect against LPS/INF-γ induced damage, in all cells object of the study (**A**–**D**). Data are representative of at least three independent experiments. *** *p* < 0.001 versus CTR ^#^ *p* < 0.05, ^##^ *p* < 0.01 and ^###^ *p* < 0.001 versus LPS/ INF-γ.

The potential of PEA-um in mediating neuroinflammation is known [4], but the in vitro role of PEA-um in modulating the action of neuronal and non-neuronal cells involved in inflammatory processes, such as astrocytes and microglia, has never been investigated in detail. In this context, to induce inflammatory stimuli, all cell lines were stimulated with LPS/IFN γ and at the same time treated with different concentrations (1, 3, and 10 μM) of PEA-um. The results obtained showed a significant protective effect, against LPS/IFN γ induced inflammatory process, in cells treated with PEA-um 3 and 10 µM, but not at the lowest concentration 1 µM (Figure 2A–D).

### 2.2. Effects of NAAA Silencing in Neuronal and Non-Neuronal Cells

**Figure 3:**
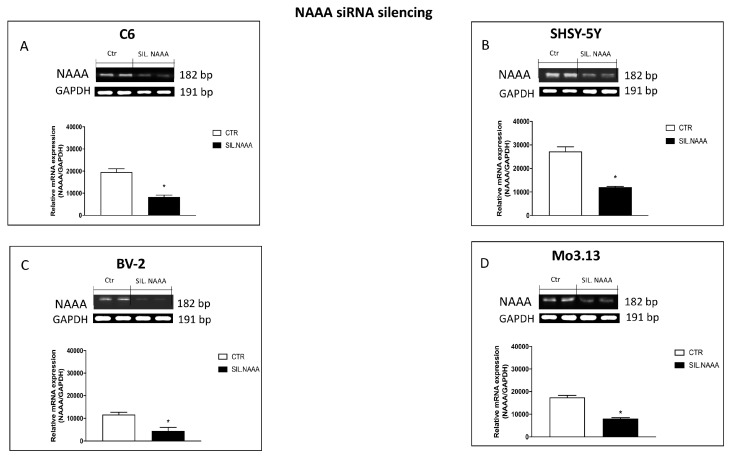
Evaluation of *N*-acylethanolamine acid amidase (NAAA) expression following NAAA small interfering RNA (siRNA) knockdown. NAAA mRNA expression was determined in C6, SHSY-5Y, BV-2, and Mo3.13 cells, that were either transfected for 48 h with NAAA-specific siRNA. Values are normalized to GAPDH and expressed as fold change to untreated control cells (**A**–**D**). The silencing efficiency was achieved in all cell lines studied (**A**–**D**). Data are representative of at least three independent experiments. (**A**) * *p* < 0.05 versus CTR; (**B**) * *p* < 0.05 versus CTR; (**C**) * *p* < 0.05 versus CTR; (**D**) * *p* < 0.05 versus CTR.

NAAA enzymes, in terms of substrates, when assessed in vitro, has a strong preference for saturated NAEs, with PEA being the preferred substrate [15]. About that, an increasing number of studies have described the synthesis and pharmacological characterization of NAAA inhibitors leading to the development of potent and stable inhibitors that enable to study the effects of NAAA inhibition in preclinical disease models, notably in the context of inflammation [16]. However, though pharmacological blockade of receptors constitutes a reliable and widely used method, silencing of NAAA constitutes a matter of interesting perspective to completely rule out their implication in the protective effects produced by the treatments. For this reason, quantitative measurements of NAAA mRNA expression was performed in all cell lines to evaluate the success of NAAA silencing. The results indicated that the relative mRNA levels of NAAA enzyme were significantly reduced in all cell lines subjects of study: C6, SHSY-5Y, BV-2, and Mo3.13 cell lines (respectively, Figure 3A–D), suggesting a significant reduction in the amount of endogenous PEA degraded and therefore an increase in available endogenous PEA.

### 2.3. Synergistic Role of PEA-um Treatment And NAAA Silencing in Countering In Vitro Neuroinflammation 

NAEs inactivation (AEA, OEA, and PEA) occurs essentially by enzymatic hydrolysis by NAAA and it is known that PEA-um administration reduces this degradation process by substrate competition [17]. Moreover, it is also believable that the increased levels of endogenous PEA upon PEA-um administration have an impact on the expression of NAAA hydrolytic enzyme [18]. In light of this, to corroborate that the anti-neuroinflammatory effects of PEA-um are enhanced by NAAA silencing, we evaluated the expression of inflammatory markers as iNOS and COX-2 on C6, SHSY-5Y, BV-2, and Mo3.13 cells following NAAA siRNA knockdown and treatment with PEA-um. 

**Figure 4:**
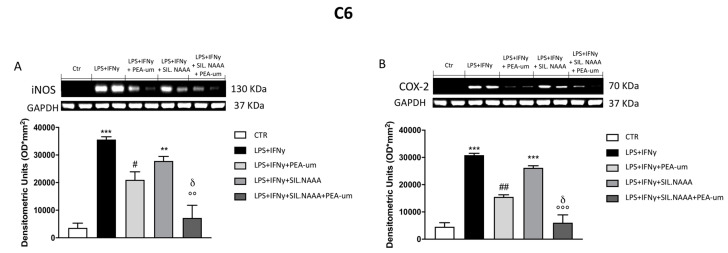
Effect of PEA-um treatment and NAAA-siRNA knockdown on the expression of iNOS and cyclooxygenase 2 (COX-2) in C6 rat glioma cells. iNOS and COX-2 levels were increased in LPS/INF-γ group, whereas treatment with PEA-um at the concentration of 10 μM significantly reduced these expressions (**A**,**B**). Blots revealed a significant increase of iNOS and COX-2 in LPS/INF-γ group, in which the cells were NAAA-silenced (**A**,**B**); meanwhile, their expression was attenuated in group treated with PEA-um 10 μM (**A**,**B**). Data are representative of at least three independent experiments. ** *p* < 0.01 and *** *p* < 0.001 versus Ctr; ^#^ *p* < 0.05 and ^##^ *p* < 0.01 versus LPS/INF-γ; °° *p* < 0.01 and °°° *p* < 0.001 versus LPS/INF-γ + silenced NAAA; δ *p* < 0.05 versus LPS/INF-γ + NAAA silencing + PEA-um treatment.

**Figure 5:**
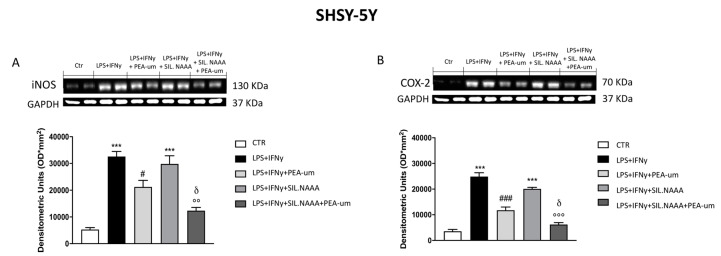
Effect of PEA-um treatment and NAAA-siRNA knockdown on the expression of iNOS, and COX-2 in SHSY-5Y human neuroblastoma cells. An increase in iNOS and COX-2 expression was observed in LPS/INF-γ group, significantly reduced by treatment with PEA-um at the concentration of 10 μM (**A**,**B**). Blots revealed a significant increase of iNOS and COX-2 in LPS/INF-γ group, in which the cells were NAAA-silenced (**A**,**B**); treatment with PEA-um 10 μM significantly reduced inflammatory markers expression in cells NAAA-silenced compared to only silenced cells or to only PEA-um treated cells (**A**,**B**). Data are representative of at least three independent experiments. *** *p* < 0.001 versus Ctr; ^#^ *p* < 0.05 and ^###^ *p* < 0.001 versus LPS/INF-γ; °° *p* < 0.01 and °°° *p* < 0.001 versus LPS/INF-γ + silenced NAAA; ^δ^ *p* < 0.05 versus LPS/INF-γ + NAAA silencing + PEA-um treatment.

**Figure 6:**
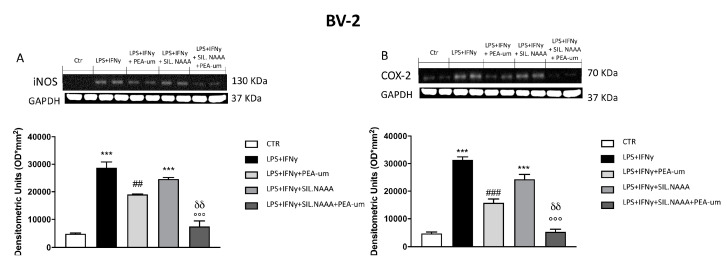
Effect of PEA-um treatment and NAAA-siRNA knockdown on the expression of iNOS, and COX-2 in BV-2 mouse microglial cells. The stimulation with LPS/INF-γ significantly increased iNOS and COX-2 expression, compared to control (**A,B**). Treatment with PEA-um at the concentration of 10 μM significantly reduced these expressions (**A,B**). In NAAA-silenced cells, stimulated with LPS/INF-γ, the expression of iNOS and COX-2 significantly increased compared to a notably reduction following PEA-um treatment (**A**,**B**). Data are representative of at least three independent experiments. *** *p* < 0.001 versus Ctr; ^##^ *p* < 0.01 and ^###^ *p* < 0.001 versus LPS/INF-γ; °°° *p* < 0.001 versus LPS/INF-γ + silenced NAAA; ^δδ^ *p* < 0.01 versus LPS/INF-γ + NAAA silencing + PEA-um treatment.

**Figure 7:**
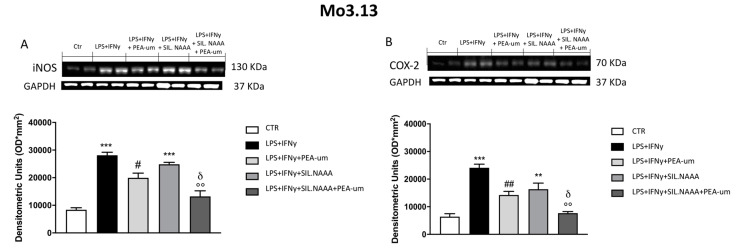
Effect of PEA-um treatment and NAAA-siRNA knockdown on the expression of iNOS, and COX-2 in Mo3.13 human oligodendrocytes. An increase in iNOS and COX-2 expression was observed in LPS/INF-γ group, significantly reduced by treatment with PEA-um 10 μM (**A**,**B**). NAAA silencing significantly increase inflammatory markers expression, while PEA-um 10 μM treatment counteracts the inflammatory process (**A**,**B**). Data are representative of at least three independent experiments. ** *p* < 0.01 and *** *p* < 0.001 versus Ctr; ^#^ *p* < 0.05 and ^##^ *p* < 0.01 versus LPS/INF-γ; °° *p* < 0.01 versus LPS/INF-γ + silenced NAAA; ^δ^ *p* < 0.05 versus LPS/INF-γ + NAAA silencing + PEA-um treatment.

In rat C6 glioma cells, treatment with PEA-um (10 μM) significantly reduced iNOS and COX-2 expression following damage induced by LPS/IFN γ (Figure 4A,B), but the protective effects of PEA-um were enhanced in LPS/IFN γ-stimulated cells in which NAAA enzyme was muted (Figure 4A,B). The same condition was observed in human SH-SY5Y, with a remarkable PEA-um protection in LPS/IFN γ-stimulated cells (Figure 5A,B), but, in this case, iNOS and COX-2 expression was even more reduced in treated cells in which NAAA was silenced, compared to not NAAA-silenced SHSY-5Y (Figure 5A,B). Activated microglia is able to secrete a number of pro-inflammatory and neurotoxic factors which cause neuronal damage [19]; in this experiment, we confirmed the expression of inflammatory markers iNOS and COX-2 in microglia BV-2 cells following LPS/IFN γ-activation (Figure 6A,B), with a notably reduction when the cells were treated with PEA-um (10 μM); an enhancement of the effect in the PEA-um treated cells was obtained with the NAAA-siRNA knockdown, compared to cells only treated or only NAAA-silenced (Figure 6A,B). Furthermore, also in Mo3.13 oligodendrocytes cell lines, COX-2 expression, more than iNOS protein levels, was significantly reduced in PEA-um (10 μM)-treated cells, compared to only stimulated cells (Figure 7A,B); the NAAA—siRNA knockdown significantly reduced the expression of both inflammatory markers, with an enhanced effect in PEA-um treated NAAA-silenced cells (Figure 7A,B).

## 3. Discussion

Inflammation is a key element in the pathobiology of chronic pain, neurodegenerative diseases, stroke, and neuropsychiatric disorders; glial cells, key players in nervous system disorders, respond to inflammatory signals of immune origin [4]. Communication between the nervous system and immune system represents a fundamental principle underlying neuroinflammation; particularly, immune cell-derived inflammatory molecules are critical for regulation of responses to inflammation; these mediators can originate from various sources.

Neuroinflammation responds to a program of resolution that involves lipid mediators endowed with the capacity to switch off inflammation process. These naturally occurring lipid signaling molecules include the NAEs, specifically PEA. PEA may play a role in maintaining cellular homeostasis, in mast cell-mediated models of neurogenic inflammation and neuropathic pain and it is neuroprotective in in vivo models of stroke, spinal cord injury, traumatic brain injury, and neurodegenerative diseases [20,21]. Moreover, PEA in ultra-micronized form, known as PEA-um, shows superior efficacy in inflammatory models when compared to naïve PEA. Marked properties in peripheral inflammation models emerge by PEA administration, correlated to high effectiveness in a number of neurodegenerative disorders, including AD, Parkinson’s disease, and multiple sclerosis [22,23].

NAEs are hydrolyzed to the corresponding fatty acid and ethanolamine by specific hydrolase and amidase, respectively, FAAH and NAAA [24,25]. In contrast to FAAH, NAAA hydrolyzes NAEs having less than 18 carbon atoms, i.e., PEA [26]. Inhibiting the enzymatic degradation of PEA by targeting NAAA, in principle, represents another route in treatment of neuroinflammation; for this, a number of selective NAAA inhibitors have been described including systemically active compounds which are able to modulate responses induced by inflammatory stimuli in vivo and in vitro [2,27], although the validity of this approach remains to be demonstrated. 

PEA is produced on demand and its catabolic enzymes are proposed to modulate substrate availability; so, a modulatory approach intended for inhibit PEA degradation and make the exogenous PEA more available through ultra-micronization would maximize accessibility of the NAE’s component molecules in biological system.

Given the complex nature of non-neuronal cellular involvement in inflammation-associated pathologies across the CNS and PNS, viewing neuroinflammation in the context of microglia astrocyte or mast cell involvement allows us to appreciate the homotypic and heterotypic cell–cell interactions that are an integral part of the inflammation process [28]. On this basis, the aim of the research was to perform an in vitro study of neuroinflammation on different types of neuronal and non-neuronal cell lines: C6 glioma cells, SHSY-5Y neuroblastoma cell line, BV-2 microglia cells, and Mo3.13 oligodendrocytes, together silencing NAAA enzyme and treating with PEA-um, thus providing a greater amount of PEA needed to counteract inflammation triggered at cellular level.

Neuroinflammation is linked to synapse loss and cognitive decline both in humans and in pre-clinical models, but important questions remain about the cellular mechanisms that existing experimental systems cannot easily address [29]. Lipopolysaccharide (LPS), a cell-wall immunostimulatory component of gram-negative bacteria, once activated, produces proinflammatory cytokines that are key mediators of the neuro-inflammatory process [29,30,31] The immunoregulatory properties of PEA, as an anti-inflammatory agent, are better known in vivo studies [32]. In the first time, in this work, the protective activity of PEA-um, at three different concentrations, was evaluated in counteracting the in vitro inflammation induced in the different cell lines stimulated with LPS and IFN γ. This study demonstrated, for the first time, that PEA-um, at the highest concentrations of 3 and 10 μM, significantly counteracted LPS-induced inflammation, as well as increased cell viability in glioma and glial cells LPS/IFN γ-damaged.

PEA availability into the cells depends on its degradation and deactivation, that preferentially happens by a lysosomal enzyme NAAA [14]. NAAA hydrolyzes PEA to palmitic acid and ethanolamine, with much greater efficacy and selectivity than FAAH, so inhibition of NAAA is a good strategy to target inflammation by modulating the tissue levels of PEA. However, recently NAAA inhibitors have begun to emerge, and most of them suffer from the chemical and biological unstable properties, which restrict functions of NAAA inhibition [33]. For the first time, in this research, the inhibition of NAAA expression was performed through genetic silencing of the enzyme in C6, SHSY-5Y, microglia, and oligodendrocytes cells. Using this novel, more potent, and selective method, it was possible to unravel in detail the role of NAAA activity and PEA disposability in mediating the inflammatory process.

Although the inflammatory response is a defense mechanism against injury, sustained inflammation is a pathological condition, which determines an over-expression of several pro-inflammatory enzymes and reactive species; particularly, iNOS being up-regulated during the inflammatory process [34]. Other important mediators over-expressed during inflammation are prostaglandins (PGs), bioactive signaling molecules derived from cyclooxygenase (COX) [35]. 

The up-regulation of iNOS and COX-2 during inflammation is controlled by the pro-inflammatory transcription factor NF-κB [34]. PEA prevents NF-κB nuclear translocation, confirming the involvement of this transcriptional factor as interesting tools for inflammatory control [36]. For the first time, in this study, we demonstrated that treatment with PEA-um, at the highest concentration of 10 μM, significantly reduced inflammation as showed by a decreased expression in iNOS and COX-2 protein levels, in glioma and in neuroblastoma cells, as well as in microglia and oligodendrocytes, compared to cells stimulated but not treated. Main actors in inflammatory scenario, together with neuronal cells, are astroglia and microglia; upon activation, also called “gliosis,” these cells get involved in the production of cytokines and chemokines, which maintain and enhance the inflammatory condition [37]. The biological meaning if these experiments was how PEA-um is able to modulate inflammatory markers in the same way in different cell types (neuronal and non-neuronal). 

Moreover, to understand if the treatment with exogenous PEA-um could enhance the PEA endogenous availability through the silencing of the degrading enzyme NAAA, thus regulating the expression of inflammatory markers, all cell lines were subjected to a NAAA siRNA-knockdown. A significant reduction in iNOS and COX-2 expression was obtained in NAAA-silenced cells, constantly in the different cell lines, compared to control. The amazing discovery, however, was to observe a significant reduction in inflammatory markers in NAAA-silenced cells treated exogenously with PEA-um, suggesting as the lack of NAAA empowered PEA protective effect, in diversified cellular context: neuronal and non-neuronal.

The data obtained, highlighted as in vitro silencing of intracellular NAAA, associated to a supply of exogenous PEA, made more available in the ultra-micronized form (PEA-um), could represent a novel therapy to control cellular neuroinflammation.

## 4. Materials and Methods

### 4.1. Cell Lines Culture

#### 4.1.1. C6 Cell Line

Rat C6 glioma cell line from *Rattus Norvegicus* C6, obtained from American Type Culture Collection (ATCC^®^ CCL-107™, ATCC, Manassas, VA, USA), was cultured in 75-cm^2^ flasks in Dulbecco’s modified essential medium (DMEM) with 10% fetal bovine serum (FBS), 2 mM L-glutamine, 100 U/mL penicillin, and 100 μg/mL streptomycin at 37 °C under 5% CO_2_ humidified air. Cells were passaged at confluence using a solution of 0.025% trypsin and 0.01% EDTA. 

#### 4.1.2. SH-SY5Y Cell Line

SH-SY5Y cells, obtained from American Type Culture Collection (ATCC CLR-2266, ATCC), are a cloned subline of SK-N-SH cells originally established from a bone marrow biopsy of a neuroblastoma patient [38]. SH-SY5Y neuroblastoma cells can be differentiated into neuron-like cells displaying morphological and biochemical features of mature neurons. Neuronal differentiation was achieved using retinoic acid (RA) as previously described [39]; differentiation of cell lines into neuronal-like cell lines is required to mimic the intracellular environment of a neuronal cell. SH-SY5Y cells were grown to monolayer in 75-cm^2^ flasks containing DMEM and Ham’s F12, modified with 2 mmL-glutamine, 1.0 mM sodium pyruvate, and supplemented with FBS to 10%, streptomycin 50 μg/mL. SH-SY5Y cells were maintained at 37 °C and 5% CO_2_. Cells were passaged at confluence using a solution of 0.025% trypsin and 0.01% EDTA.

#### 4.1.3. BV-2 Cell Line

BV-2 mouse cell line is a brain microglial cell line from C57BL/6, obtained from Cell bank IRCCS AOU San Martino IST (Genova, Italy) Interlab Cell Line Collection (ICLC) (ICLC ATL03001). This cell line exhibits morphological, phenotypical, and functional properties of microglial cells; secretory and effector functions, also ascribed to microglia, have been extensively characterized in this cell line [40]. BV-2 cells were maintained in medium consisting of equal volumes of minimum essential medium (MEM) and DMEM supplemented with 5% heat inactivated fetal calf serum (FCS), 100 IU/mL penicillin G, and 100 μg/mL streptomycin in a humidified 5% CO2 atmosphere at 37 °C. Cells were passaged at confluence using a solution of 0.025% trypsin and 0.01% EDTA. 

#### 4.1.4. Mo3.13 Cell Line

Mo3.13, human glial (oligodendrocytic) hybrid cells, obtained from Cedarlane Laboratories (Cedarlane Labs, Burlington, Canada) (CLU301-P) were cultured in 75-cm^2^ flasks containing DMEM supplemented with 10% FCS, 100 U/mL penicillin, and 100 µg/mL streptomycin in a 5% CO2 incubator at 37 °C. Differentiated cells were prepared by first washing 70% confluent cells in DMEM-lacking serum and then culturing in DMEM supplemented with 100 U/mL penicillin, 100 µg/mL streptomycin, and 100 nM 4-^N^_L_-phorbol 12-myristate 13-acetate (PMA) for 3–4 days prior to an experiment.

### 4.2. Experimental Procedures

All different types of cells were cultured for 3 days to confluence in complete medium. Specifically, for cell viability, 4 × 10^4^ cells were plated in 96-well plates (Corning Cell Culture, Corning, NY, USA) in a volume of 150 μL. Progressive dilutions of PEA-um (1–3–10 μM) were used to establish the high concentrations with less toxicity on cell viability, using 3-(4,5-dimethylthiazol-2-yl)-2,5-diphenyltetrazolium bromide (MTT) colorimetric assay, as previous described [41]. 

After 24 h of starvation in serum-free DMEM/F12, cells were stimulated for 24 h with LPS (1 μg/mL) and INF-γ (100 U/mL) in the presence or absence of PEA-um (1–3–10 μM).

All cell lines were divided in the following experimental groups:

Control (CTR), cells were cultured with normal culture medium;

LPS, cells were stimulated with LPS (1 μg/mL) and INF-γ (100 U/mL);

LPS+PEA-um (1 μM), cells were stimulated with LPS (1 μg/mL) and INF-γ (100 U/mL) for 24 h and treated with PEA-um (1 μM);

LPS+PEA-um (3 μM), cells were stimulated with LPS (1 μg/mL) and INF-γ (100 U/mL) for 24 h and treated with PEA-um (3 μM);

LPS+PEA-um (10 μM), cells were stimulated with LPS (1 μg/mL) and INF-γ (100 U/mL) for 24 h and treated with PEA-um (10 μM).

### 4.3. Cell Viability Assay (MTT Assay)

The cellular viability of different cell lines was assessed using a mitochondria-dependent dye for live cells (tetrazolium dye; MTT) to formazan. Cultures were treated with three different concentrations (1, 3, 10 μM) of PEA-um for 24 h and then incubated at 37 °C with MTT (0.2 mg/mL) for 1 h. The medium was removed and the cells lysed with dimethyl sulfoxide (DMSO) (100 µL), as previously described [41]. The extent of reduction in MTT to formazan was quantified by measurement of optical density at 550 nm with a microplate reader.

### 4.4. Small Interfering RNA (siRNA) Transfection and Polimerase Chain Reaction (PCR)

Cells were transfected with 20 nM siRNA against NAAA or 20 nM control siRNA (Qiagen, Hilden, Germany) for 48 h using Lipofectamine RNAi-MAX transfection reagent (Life Technologies, Milan, Italy) following the manufacturer’s instructions as previously described [41]. Total RNA (2 μg) isolated from C6, SH-SY5Y, BV-2, and Mo3.13 (4.5 × 10^5^ cells on 6-well plates) was reverse transcribed and synthesized complementary DNA (cDNA )was used as a template for PCR. RT-PCR was performed on a T100 Thermal Cycler (Bio-Rad, Hercules, CA, USA) with Taq polymerase (Life Technologies). cDNAs underwent 30 cycles for NAAA, each one performed at 94 °C for 1 min, melting temperature (Tm) °C for 45 s and 72 °C for 55 s. After this treatment, 10 μL of RT-PCR products was separated by 1.5% agarose gel electrophoresis in Tris/Borate/EDTA (TBE) 0.5 (Tris-base 0.089 M, boric acid 0.089 M) containing ethidium bromide. Fragments of DNA were seen under ultraviolet light. The primer sets were used to detect specific PCR products, and their values were calculated as fold change relative to control after normalization to the *GAPDH* gene. The human primer for NAAA was: 5′-TTAAGCTTGAGCCCGAGCC-3′ (sense) and 5′-TCCTCGAGGATCCTTTCTACTCGGGTTTCT-3′ (antisense); the mouse primer for NAAA was 5′-AAGGCTGGTGGTGGGAGAA-3′ (sense) and 5′-TCAGCAATGAGGGGAGTCTTG-3′ (antisense).

### 4.5. Western Blot Analysis

For Western Blot analysis, 5 × 10^5^ cells were plated in 6-well plates (Corning Cell Culture), stimulated for 24 h with LPS/ IFN-γ (1 μg/mL and 100 U/mL, respectively) in presence of PEA-um 10 μM, washed with phosphate buffered saline (PBS), scraped, and pelleted for protein lysates preparation, as previously described [42]. C6, SH-SY5Y, BV-2, and Mo3.13 cell lines were washed two times with ice-cold phosphate buffered saline (PBS), harvested, and resuspended in Tris-HCl 20 mM pH 7.5, NaF 10 mM, 150 mM NaCl, 1% Nonidet P-40, and protease inhibitor cocktail (Roche, Basel, Switzerland). After 40 min, cell lysates were centrifuged at 16,000× *g* for 15 min at 4 °C. Protein concentration was estimated by the Bio-Rad protein assay using bovine serum albumin as standard. Samples were heated at 95 °C for 5 min, and the same amounts of protein separated on 12% SDS-PAGE gel and blotted to a PVDF membrane (Immobilon-P). Membranes were incubated overnight at 4 °C with the following primary antibodies anti-inducible nitric oxide synthase iNOS (1:1000 BD transduction), anti-cyclooxygenase 2 (COX-2) (1:500; Santa Cruz Biotechnology, Dallas, TX, USA). Membranes were then incubated with peroxidase-conjugated bovine anti-mouse secondary antibody or peroxidase-conjugated goat anti-rabbit IgG (1:2000, Jackson ImmunoResearch, Jackson Laboratories Bar Harbor, ME, USA) for 1 h at room temperature. To ascertain that blots were loaded with equal amounts of proteins they were also incubated in the presence of the antibody against β-actin protein for cytosolic fraction (1:500; Santa Cruz Biotechnology) and Lamin A/C for nuclear fraction (1:500; Santa Cruz Biotechnology). The signals were detected with a chemiluminescence detection system reagent according to the manufacturer’s instructions (Super Signal West Pico Chemiluminescent Substrate, Pierce Thermo Scientific, Rockford, IL, USA). The relative expression of the protein bands was quantified by densitometry with BIORADChemi, (BIORAD, Genzano di Roma, Roma, Italy) using ImageLab software.

### 4.6. Materials

Ultra-micronized Palmitoylethanolamide was kindly provided from Epitech Group SpA (Saccolongo, Padova, Italy). Dulbecco’s modified Eagle medium (DMEM), fetal bovine serum (FBS), penicillin–streptomycin (PS), TRIZOL, oligo-dT, and Superscript II were purchased from Gibco-BRL (Gaithersburg, MD, USA). Unless otherwise stated, all compounds were acquired from Sigma Aldrich (Saint Louis, MO, USA). All other chemicals were of the highest commercial grade available. All stock solutions were prepared in non-pyrogenic saline (0.9%NaCl, Baxter, Milan, Italy).

### 4.7. Statistical Analysis

Data are presented as mean ± SEM values of three independent determinations. All experiments were done at least three times, each time with three or more independent observations. Statistical analysis was performed by One-way ANOVA test, followed by multiple comparisons performed by Bonferroni’s test. A value of *p* < 0.05 was considered significant.
